# Extensor carpi ulnaris tendon pathology and ulnar styloid bone marrow edema as diagnostic markers of peripheral triangular fibrocartilage complex tears on wrist MRI: a case–control study

**DOI:** 10.1007/s00330-023-09446-x

**Published:** 2023-02-21

**Authors:** Mika T. Nevalainen, Adam C. Zoga, Michael Rivlin, William B. Morrison, Johannes B. Roedl

**Affiliations:** 1 Department of Diagnostic Radiology, Oulu University Hospital, P.O. Box 50, 90029 Oulu, Finland; 2 Research Unit of Medical Imaging, Physics and Technology, Faculty of Medicine, University of Oulu, POB 5000, FI-90014 Oulu, Finland; 3 Division of Musculoskeletal Imaging and Intervention, Department of Radiology, Thomas Jefferson University Hospitals, Sidney Kimmel Medical College at Thomas Jefferson University, 132 South 10th Street, Philadelphia, PA 19107 USA; 4 Department of Hand and Orthopaedic Surgery, Rothman Institute of Orthopaedics, Sidney Kimmel Medical College, Thomas Jefferson University, 925 Chestnut Street, 5th Floor, Philadelphia, PA 19107 USA

**Keywords:** Bone marrow, Magnetic resonance imaging, Triangular fibrocartilage, Tears, tendons, Wrist

## Abstract

**Objectives:**

To evaluate extensor carpi ulnaris (ECU) tendon pathology and ulnar styloid process bone marrow edema (BME) as diagnostic MRI markers for peripheral triangular fibrocartilage complex (TFCC) tears.

**Methods:**

One hundred thirty-three patients (age range 21–75, 68 females) with wrist 1.5-T MRI and arthroscopy were included in this retrospective case–control study. The presence of TFCC tears (no tear, central perforation, or peripheral tear), ECU pathology (tenosynovitis, tendinosis, tear or subluxation), and BME at the ulnar styloid process were determined on MRI and correlated with arthroscopy. Cross-tabulation with chi-square tests, binary logistic regression with odds ratios (OR), and sensitivity, specificity, positive predictive value, negative predictive value, and accuracy were used to describe diagnostic efficacy.

**Results:**

On arthroscopy, 46 cases with no TFCC tear, 34 cases with central perforations, and 53 cases with peripheral TFCC tears were identified. ECU pathology was seen in 19.6% (9/46) of patients with no TFCC tears, in 11.8% (4/34) with central perforations and in 84.9% (45/53) with peripheral TFCC tears (*p* < 0.001); the respective numbers for BME were 21.7% (10/46), 23.5% (8/34), and 88.7% (47/53) (*p* < 0.001). Binary regression analysis showed additional value from ECU pathology and BME in predicting peripheral TFCC tears. The combined approach with direct MRI evaluation and both ECU pathology and BME yielded a 100% positive predictive value for peripheral TFCC tear as compared to 89% with direct evaluation alone.

**Conclusions:**

ECU pathology and ulnar styloid BME are highly associated with peripheral TFCC tears and can be used as secondary signs to diagnose tears.

**Key Points:**

• *ECU pathology and ulnar styloid BME are highly associated with peripheral TFCC tears and can be used as secondary signs to confirm the presence of TFCC tears*.

• *If there is a peripheral TFCC tear on direct MRI evaluation and in addition both ECU pathology and BME on MRI, the positive predictive value is 100% that there will be a tear on arthroscopy compared to 89% with direct evaluation alone*.

• *If there is no peripheral TFCC tear on direct evaluation and neither ECU pathology nor BME on MRI, the negative predictive value is 98% that there will be no tear on arthroscopy compared to 94% with direct evaluation alone*.

## Introduction

One of the most common causes of ulnar-sided wrist pain is an injury to the triangular fibrocartilage complex (TFCC), which acts as a stabilizer of the distal radioulnar and ulnocarpal joints and functions to distribute compressive forces during axial loading. The TFCC is composed of the triangular fibrocartilage, meniscal homologue, dorsal and volar radioulnar ligaments, ulnolunate ligament, ulnotriquetral ligament, and the subsheath of the extensor carpi ulnaris (ECU) tendon [1]. The TFCC consists of three components, a central component (articular disc proper), a radial component which attaches to the sigmoid notch of the radius, and an ulnar component which attaches with two limbs to the ulna. As an addon to this complex anatomy, the peripheral TFCC blends at its ulnar attachments with the ECU tendon subsheath. The ECU subsheath is band of tissue overlying the ECU tendon spanning the ulnar groove; it is the primary restraint of ECU subluxation by keeping the ECU tendon in the ulnar groove. The most commonly applied classification system for TFCC tears is the Palmer classification, which divides the tears to traumatic (type 1) and degenerative injuries (type 2) [2]. However, even though widely used with high reproducibility [3], this classic system lacks detail in modern radiologic setup; for instance, the classification does not account for the morphologic features (capsular injuries, bucket handle, or horizontal tears) or combined tears [4].

Although MR arthrography (MRA) has been long considered superior to conventional MRI [5], the recent studies show that routine MRI also comes with high diagnostic accuracy for TFCC tears [4, 6, 7]. Despite the modern MRI techniques, certain TFCC tears remain difficult to visualize on MRI. For instance, peripheral tears at the ulnar attachment of the TFCC pose a diagnostic challenge in everyday practice. It is essential that TFCC tears are reported meticulously since there is a significant difference in terms of management decisions between central perforations and peripheral tears. Based on the close anatomic relationship between the peripheral ulnar TFCC and the ECU tendon, we hypothesize that peripheral TFCC tears are associated with ECU tendon pathologies. Additionally, our division has anecdotally noted over the years that peripheral TFCC tears on MRI are often seen together with subtendinous bone marrow edema (BME) at the ulnar styloid underlying the transitional zone of the peripheral ulnar TFCC and the ECU subsheath. Therefore, we hypothesize that local BME in addition to ECU pathology might be used as a secondary diagnostic marker on MRI indicating a peripheral TFCC injury.

## Materials and methods

### Study design and patients

Institutional review board approval was obtained and the requirement for informed consent was waived. The study is compliant with the Health Insurance Portability and Accountability Act (HIPAA). Consecutive patients were enrolled to this retrospective investigation between October 2008 and July 2017 from an orthopedic hand surgery clinic (Rothman Institute of Orthopaedics, Thomas Jefferson University, Philadelphia, USA) for this case–control study. The patients suffered from ulnar-sided wrist pain due to both traumatic and non-traumatic causes. Inclusion criteria were wrist MRI performed within 6 weeks prior the arthroscopy, arthroscopic correlation evaluating the TFCC, and subject’s age ≥ 18 years. Patients with history of inflammatory arthritis or previous surgery were excluded.

### MRI, image analysis, and wrist arthroscopy

MR images were performed at two 1.5-T MRI scanners (General Electric Optima) with dedicated wrist coils. The standard wrist protocol with 2D coronal T1, coronal PD fat-saturated, coronal GRE fat-saturated, axial PD fat-saturated and sagittal STIR sequences were used. The MR images were interpreted by two experienced fellowship-trained musculoskeletal radiologists with 6 and 15 years of experience on musculoskeletal radiology, and in case of discordance, a third senior musculoskeletal radiologist made the final decision.

**Fig. 1:**
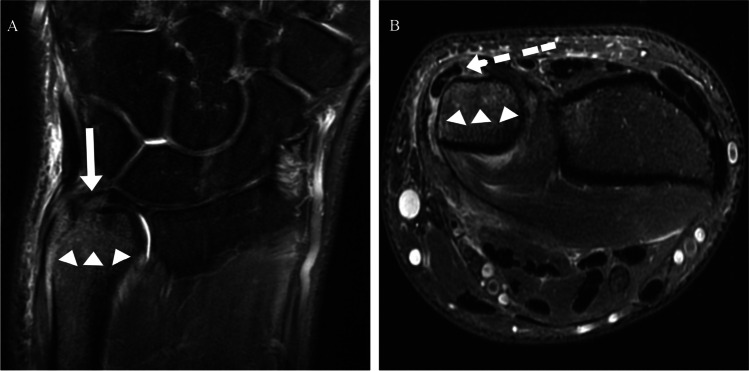
Coronal (**A**) and axial (**B**) T2-weighted fat-saturated sequences demonstrating a peripheral TFCC tear (white arrow) with ECU tendon subluxation and tenosynovitis (dashed white arrow), and distinct ulnar styloid BME (white arrowheads)

**Fig. 2:**
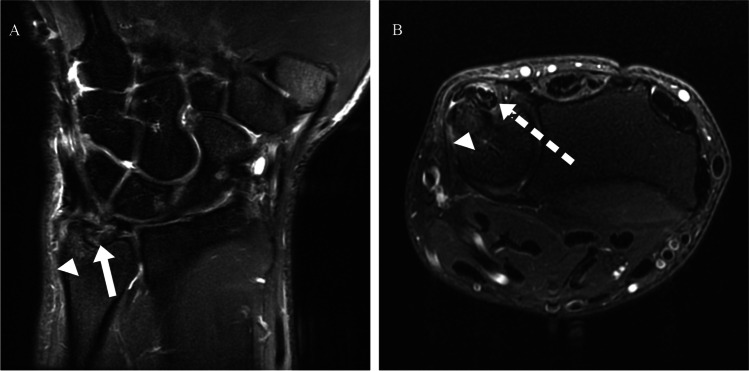
Coronal (**A**) and axial (**B**) T2-weighted fat-saturated sequences showing a peripheral TFCC tear (white arrow) with marginal ECU tendon subluxation, partial intratendinous tear and tenosynovitis (dashed white arrow), and mild ulnar styloid BME (white arrowheads)

First, the TFCC was evaluated for tears classified as central (at the articular disc) tears or peripheral tears (at the ulnar or at the radial attachment) [8]. Second, the ECU tendon was assessed reviewing it in all three planes: Tendinosis was defined as tendon thickening and slightly increased signal. Tendinosis was subclassified as absent or present. A partial tear was considered as fluid-bright, hyperintense signal within the tendon with disruption of fibers. A complete tear was considered when there was discontinuity of the entire tendon with a tendon gap. Fluid in the tendon sheath surrounding the tendon represents tenosynovitis, further specified as absent or present. Subluxation or luxation was reported when ECU tendon did not reside within the ulnar groove. Figures 1 and 2 show examples of ECU pathology, ulnar styloid BME, and peripheral TFCC tears on wrist MRI.

For the gold standard confirming TFCC tears, wrist arthroscopy was performed on all patients within 6 weeks after the MRI examinations, with an average interval of 23 days (range, 1–42 days). Routine arthroscopic approach was used with the patient placed in a supine position with the arm on a hand table and 10 lbs of longitudinal traction applied with the elbow at 90°. Standard diagnostic wrist arthroscopy portals were then placed: 3–4, 4–5 working, 6R, and 6U, as necessary. The dorsal arthroscopic portals are named for their relation to the extensor compartments: For the 4–5 portal, the point of entry is the interval between the extensor digitorum communis and extensor digiti minimi tendons, over the radiocarpal joint. The 6R and 6U portals are designated by their relationship, radial (R) or ulnar (U), to the ECU tendon. TFCC pathology was determined by direct observation and probing the tear when found and using adjunct provocative tests such as trampoline and suction maneuvers [9].

### Statistics

Cross-tabulation with chi-square tests was used to examine the statistical differences between two binary groups (tear types), and Kruskal–Wallis test for three binary groups. For correlation analysis, Spearman’s correlation coefficient was used. Using cross-tabulation, sensitivity, specificity, positive predictive value, negative predictive value, and accuracy were calculated to describe diagnostic efficacy of the direct evaluation of TFCC on MRI, and secondary markers (ECU pathology and BME). Confidence intervals (CIs) for sensitivity, specificity, and accuracy were calculated as exact Clopper-Pearson CIs, and for the predictive values, the standard logit confidence intervals were applied [10]. Finally, binary logistic regression with odds ratios (OR) was performed to analyze whether ECU pathology and BME could help to predict peripheral TCFC tears. SPSS software (version 28) was used and *p* < 0.001 was deemed as a statistically significant finding.

## Results

A total of 133 patients with MRI and arthroscopy (age range 21–75, average 44 years; 68 females, 65 males) were included in this study (Table 1). Out of these patients, 46 patients had no TFCC tear, 34 had a central TFCC perforation, and 53 had peripheral TFCC tears. When applying wrist arthroscopy as the gold standard for TFCC tears, ECU pathology was significantly associated with peripheral TFCC tears: ECU pathology was seen in 19.6% (9/46) of patients with no TFCC tears, in 11.8% (4/34) with central TFCC perforations, and in 84.9% (45/53) with peripheral TFCC tears (*p* < 0.001). The respective values for ulnar styloid BME were 21.7% (10/46), 23.5% (8/34), and 88.7% (47/53) (*p* < 0.001) thus also showing good association with peripheral TFCC tears. There was only a mediocre correlation between ECU pathology and ulnar styloid BME (*r* = 0.384, *p* < 0.001). The binary regression analysis showed that there was additional value from ECU pathology (odds ratio 27.0, CI 9.9–73.2, *p* < 0.001) and BME (odds ratio 29.0, CI 11.2–75.6, *p* < 0.001) in predicting peripheral TFCC tears. Table 2 shows the diagnostic accuracy of direct MRI evaluation, and combined approaches with ECU pathology and ulnar styloid BME for peripheral TFCC tears. If there is a peripheral TFCC tear on direct MRI evaluation and in addition both ECU pathology and BME on MRI, the positive predictive value is 100% that there will be a peripheral TFCC tear on arthroscopy compared to 89% with direct evaluation alone. If there is no peripheral TFCC tear on direct MRI evaluation and neither ECU pathology nor BME on MRI, the negative predictive value is 98% that there will be no peripheral TFCC tear on arthroscopy compared to 94% with direct evaluation alone.

**Table 1:** The demographic data of the patients included within this study

Sex (male/female)	Age (mean, range)	Duration of symptoms (mean, range)^1^	Etiologic factor (trauma/overuse)^2^	ECU pathology (yes/no)	Ulnar styloid BME (yes/no)
65 (49%)/68 (51%)	44 years, (21–75 years)	7 months, (1–180 months)	58 (54%)/49 (46%)	58 (44%)/75 (56%)	65 (49%)/68 (51%)

*ECU*, extensor carpi ulnaris; *BME*, bone marrow edema

^1^Data available on 109 patients

^2^Data available on 107patients

**Table 2:** The diagnostic accuracies for peripheral TFCC tears of direct MRI evaluation and combined approaches with ECU pathology and ulnar styloid BME. The 95% confidence intervals are given within parentheses

	Direct MRI evaluation	MRI evaluation and ECU pathology and BME	MRI evaluation or ECU pathology or BME
Sensitivity	90.6% (79.3–96.9) *n* = 48/53	75.5% (61.7–86.2) *n* = 40/53	98.1% (89.9–99.9) *n* = 52/53
Specificity	92.5% (84.4–97.2) *n* = 74/80	100.0% (95.5–100.0) *n* = 40/40	61.3% (49.7–71.9) *n* = 49/80
Positive predictive value	88.9% (78.7–94.6) *n* = 48/54	100.0% (100.0–100.0) *n* = 80/80	62.7% (56.0–68.9) *n* = 52/83
Negative predictive value	93.7% (86.5–97.2) *n* = 74/79	86.0% (79.3–90.8) *n* = 80/93	98.0% (87.5–99.7) *n* = 49/50
Accuracy	91.7% (85.7–95.8) *n* = 122/133	90.2% (83.9–94.7) *n* = 120/133	75.9% (67.8–82.9) *n* = 101/133

*ECU*, extensor carpi ulnaris; *BME*, bone marrow edema; *TFCC*, triangular fibrocartilage complex

## Discussion

In this retrospective case–control study, we found that ECU pathology and ulnar styloid BME are associated with peripheral TFCC tears and can be used as secondary findings on MRI. Only a few studies in the past investigated the diagnostic value of conventional MRI for peripheral TFCC tears: Haims et al (2002) [11] (at 1.5-T MRI) reported a sensitivity of 18%, Magee et al (2009) [12] (at 3.0-T MRI) of 75%, and Lee et al (2013) [13] (at 3.0-T MRI) of 70%; the values for specificities in these three studies were 77%, 100%, and 97%, respectively. In the most recent meta-analysis, the pooled sensitivity was 71% (CI 55–84%) and specificity 98% (CI 94–99%) for diagnosing peripheral TFCC tears on MRI [5]. In a recent paper, Daunt et al (2021) [7] (using both 1.5-T and 3.0-T systems) studied 60 patients with MRI and subsequent wrist arthroscopy; MRI had 100% sensitivity, 67% specificity, and 82% accuracy for diagnosing peripheral TFCC tears. Our study revealed a sensitivity of 91%, specificity of 93%, and accuracy of 92% for MRI (direct evaluation) for diagnosing peripheral TFCC tears. Compared to the other four abovementioned studies, two studies had higher accuracies [12, 13] and two had lower accuracies [7, 11] indicating that our results were in the median. However, these five studies (including ours) also show the great variability of MRI accuracy in between institutions with the sensitivity ranging from 41% [11] to 100% [7] and specificity from 67% [7] to 100% [12]. This indicates that additional secondary findings on MRI such as ECU pathology and ulnar styloid BME can help getting more consistent results for the diagnosis of peripheral TFCC tears and can compensate for shortcomings of direct MRI evaluation.

The association between ECU pathology and TFCC injuries has been reported in the literature, but the studies are sparse: Santo et al (2018) studied 70 patients with TFCC tears and 70 controls, pointing out that ECU tenosynovitis and longitudinal ECU tears were more prevalent in TFCC tears group [14]. Zhan et al (2020) reported three patients with peripheral (Palmer 1B) TFCC injuries and additional ECU pathology but did not study the association between these findings in their small sample [4]. Verhiel et al (2020) assessed 67 patients with TFCC tears and 67 controls with MRI and arthroscopy, concluding that ECU pathology and distal radioulnar joint arthritis are associated with TFCC tears [15]. In the most recent work, Kim et al (2021) studied the ECU tendon subluxation with 297 MRI cases; out of these, 111 cases had arthroscopic evaluation of TFCC. The authors found an association between ECU subluxation and sprains of dorsal radioulnar ligaments, which are part of the TFCC [16]. To our knowledge, no previous studies have examined the association between ulnar styloid BME and peripheral TFCC tears. More importantly, no prior study has shown the diagnostic value of ECU pathology and BME on MRI for peripheral TFCC tears.

The results of our study can be directly applied to clinical practice the following way: If there is a peripheral TFCC tear on direct MRI evaluation and in addition both ECU pathology and BME is noted on MRI, the positive predictive value is 100% that there will be a peripheral TFCC tear on arthroscopy compared to 89% with direct evaluation alone. If there is no peripheral TFCC tear on direct MRI evaluation and neither ECU pathology nor BME on MRI, the negative predictive value is 98% that there will be no peripheral TFCC tear on arthroscopy compared to 94% with direct evaluation alone.

Several limitations exist within our study. First, this was a retrospective setup including patients from nearly 10 years from a single center. Second, MRI was conducted using 1.5-T scanners, and no subsequent MRA was performed. Third, no subclassification of peripheral TFCC tears into Palmer classes (on MRI or arthroscopy) was performed preventing more in-depth analyses. Fourth, although ECU pathology was initially divided into several groups, for the statistical analyses the subclassification was abandoned for the sake of simplicity, and to retain statistical power. Fifth, the hand surgeons performing the arthroscopic assessment were experienced, but some bias might have been present, since they were not blinded to MRI results.

In conclusion, our results suggest that on routine wrist MRI, ECU pathology and ulnar styloid BME are often seen together with peripheral TFCC tears. As a practical implication, they may be used as secondary signs to confirm the presence of a peripheral TFCC tear.

## Abbreviations

BME: Bone marrow edema
ECU: Extensor carpi ulnaris
MR: Magnetic resonance
MRA: Magnetic resonance arthrography
MRI: Magnetic resonance imaging
TFCC: Triangular fibrocartilage complex

## Declarations

### Guarantor

The scientific guarantor of this publication is Mika Nevalainen.

### Statistics and biometry

Dr. Marianne Haapea (expert statistician) kindly provided statistical advice for this manuscript. Additionally, two of the authors have significant statistical expertise.

### Methodology

• retrospective

• case–control study

• performed at one institution
